# Effect of Velocity and Contact Stress Area on the Dynamic Behavior of the Spinal Cord Under Different Testing Conditions

**DOI:** 10.3389/fbioe.2022.762555

**Published:** 2022-03-04

**Authors:** Chen Jin, Rui Zhu, Meng-lei Xu, Liang-dong Zheng, Hui-zi Zeng, Ning Xie, Li-ming Cheng

**Affiliations:** Key Laboratory of Spine and Spinal Cord Injury Repair and Regeneration of Ministry of Education, Orthopaedic Department of Tongji Hospital, Tongji University School of Medicine, Shanghai, China

**Keywords:** spinal cord, velocity, contact stress area, indentation, mechanical response, biomechanics

## Abstract

Knowledge of the dynamic behavior of the spinal cord under different testing conditions is critical for our understanding of biomechanical mechanisms of spinal cord injury. Although velocity and contact stress area are known to affect external mechanical stress or energy upon sudden traumatic injury, quantitative investigation of the two clinically relevant biomechanical variables is limited. Here, freshly excised rat spinal-cord–pia-arachnoid constructs were tested through indentation using indenters of different sizes (radii: 0.25, 0.50, and 1.00 mm) at various loading rates ranging from 0.04 to 0.20 mm/s. This analysis found that the *ex vivo* specimen displayed significant nonlinear viscoelasticity at <10% of specimen thickness depth magnitudes. At higher velocity and larger contact stress area, the cord withstood a higher peak load and exhibited more sensitive mechanical relaxation responses (i.e., increasing amplitude and speed of the drop in peak load). Additionally, the cord became stiffer (i.e., increasing elastic modulus) and softer (i.e., decreasing elastic modulus) at a higher velocity and larger contact stress area, respectively. These findings will improve our understanding of the real-time complex biomechanics involved in traumatic spinal cord injury.

## Introduction

Traumatic spinal cord injury (SCI) is a significant health challenge worldwide. SCI disrupts the central nervous system, often causing permanent motor and sensory deficits. Globally, almost 180,000 SCI cases are recorded annually (Devivo, 2012; Noonan et al., 2012; Fitzharris et al., 2014), leading to extensive research into the causes, prevention, and treatment of SCIs.

Due to complicated loading environments during SCI, it is difficult to accurately measure local tissue mechanical forces using animal models. In contrast, computational modeling offers an economical, efficient, and ethical method for investigating the mechanical etiology of SCI, as well as SCI prevention and treatment. Because tissue deformation and stress correlate with injury severity and neurological impairment (Scifert et al., 2002; Maikos et al., 2008a; Li and Dai, 2009; Russell et al., 2012; Khuyagbaatar et al., 2016), finite element computational modeling allows researchers to conduct controlled SCI simulations and predict internal tissue responses and associated injury severity under various conditions (Maikos et al., 2008a; Li and Dai, 2009; Russell et al., 2012). However, to accurately model the spinal cord, accurate and detailed anatomical geometry and experimental data of cord tissue are needed to correctly predict the stresses and strains produced in the spinal cord during injury. Over the past decades, numerous spinal cord models have been developed (Scifert et al., 2002; Greaves et al., 2008; Li and Dai, 2009; Khuyagbaatar et al., 2016). However, accurate experimental data to characterize the dynamic behavior of spinal cord tissue are still necessary.

Various testing methods, including tensile, compression, and shear, have been used to investigate the mechanical properties of the spinal cord (Shetye et al., 2014; Bartlett et al., 2016; Karimi et al., 2017; Jannesar et al., 2018; Yu et al., 2020). Traumatic SCI is caused by a mechanical force acting upon the spinal cord, leading to linear and rotational accelerations that occur in a mix of compression, tension, and shear. Because indentation causes tension, compression, and shear deformation fields (Lin et al., 2009), it is an ideal loading modality for obtaining material properties of spinal cord tissue for the study of the biomechanics of traumatic SCI. Currently, animal models, including rats, are widely used to study SCI (Li et al., 2018). However, to our knowledge, few studies have investigated the microscale mechanical properties of rat spinal cord tissue through indentation. Here, we used the indentation test method to characterize the mechanical response of the spinal cord under different testing conditions and to understand the effects of velocity and contact stress area on the spinal cord.

## Materials and Methods

### Specimen Preparation

Animal experiment protocols were approved by the animal welfare committee at Tongji Hospital affiliated with Tongji University, Shanghai. To avoid potential sex effects, the analyses used female adult Sprague-Dawley rats (260–280 g), aged >10 weeks, at which they are considered to have mature spinal cords (Clarke et al., 2009). To collect accurate constitutive data from mechanical experiments, normal spinal cord tissue specimens were collected in a way that limited damage to the organ. To this end, rats were killed by intraperitoneal injection of a lethal dose of pentobarbitone sodium (100 mg/kg) and permanent cessation of circulation confirmed by cardiac perfusion with ice-cold phosphate-buffered saline (PBS; pH 7.4). A dorsal laminectomy was then performed, and nerve roots were carefully severed. The spinal cord was then cut at the seventh cervical vertebra and the first lumbar vertebra, and harvested thoracic spinal cord segments were immediately soaked in PBS (4°C). The dura mater was carefully removed. Damaged cords were excluded from the study. Notably, we referred to this specimen as “spinal cord and pia-arachnoid complex (SCPC)” (Ramo et al., 2018a; Ramo et al., 2018b). Next, the specimens were oriented with the ventral surface facing up, attached on a slide using cyanoacrylate adhesive, and placed in a 100-mm-diameter culture dish and covered with PBS to prevent dehydration. The culture dish was then positioned under the indenter tip and the specimen equilibrated for 10 min before indentation testing. To limit proteolysis and necrosis, indentation tests were done at room temperature (24°C) within an hour of the death of the rats, as post-mortem time can greatly affect the mechanical properties of biological tissues (Bilston and Thibault, 1995).

### Indentation Setup

**FIGURE 1:**
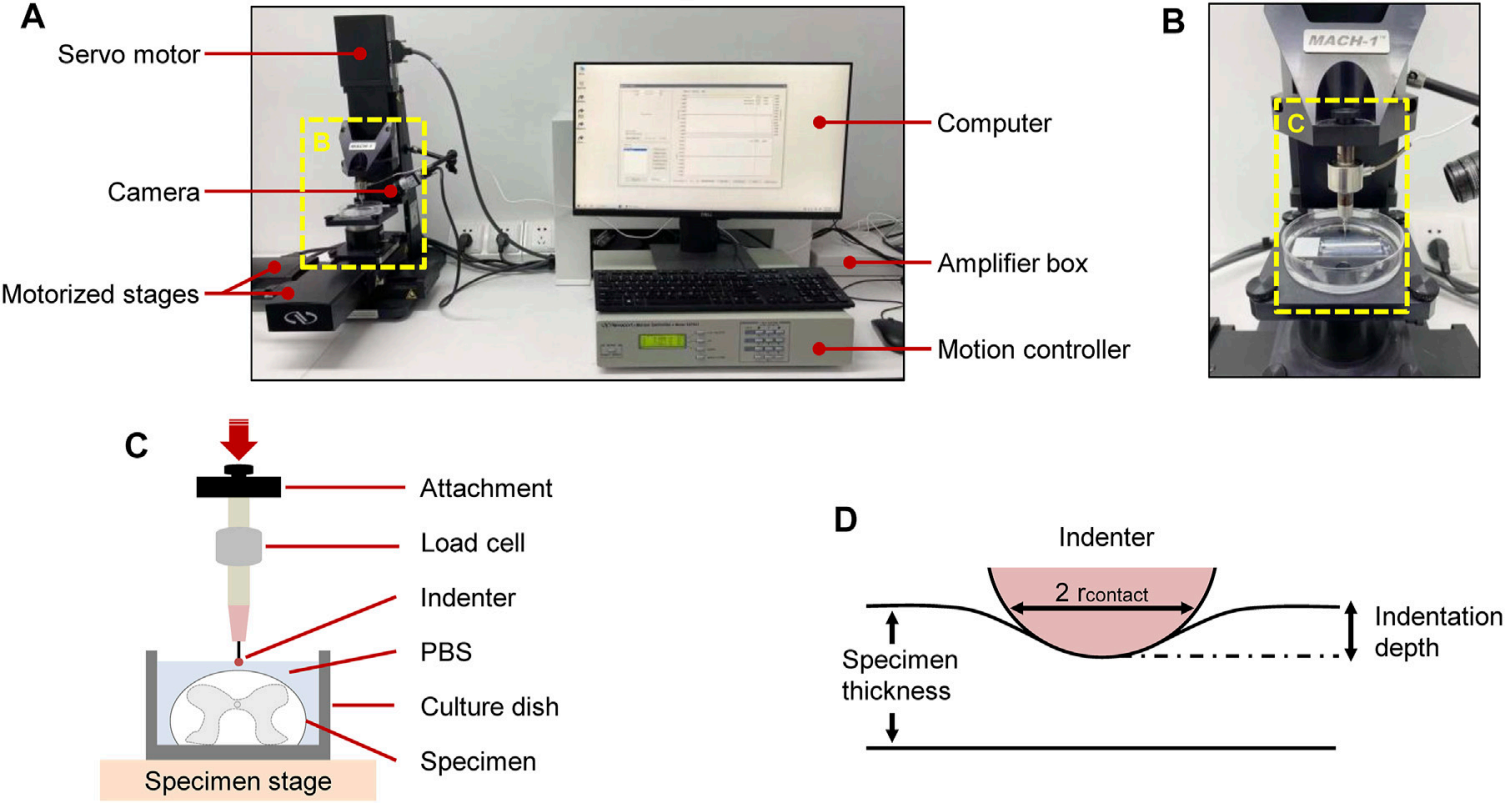
Experimental setup for *ex vivo* SCPC tissue indentation testing. Test apparatus showed indentation testing rig and assistive devices **(A)**. Mechanical indentation was conducted by a Mach-1 Model V500css test device **(B)**. Schematic of indentation testing setup **(C)**. Schematic of indentation process **(D)**.

A schematic of the setup of indentation load–relaxation tests on SCPC tissues is shown in Figure 1. Mechanical properties were measured *ex vivo* using a Mach-1 Model V500css device (Biomomentum Inc., Laval, QC, Canada). The technique precisely assessed surface orientation at each position and recorded normal load with a single-axial load cell (1.5 N range and 0.07 mN resolution on the vertical axis). The Mach-1 micromechanical system was made of the tester frame, three motorized stages, one motion controller, one load cell, one load cell amplifier, one computer, and accessories such as testing chambers and fixtures. The load cell amplifier powered the load cell and converted the measured force signal into a digital value that was relayed to a computer. The stage was commanded using a motion controller, which was in turn controlled by the Mach-1 Motion software.

### Indentation Protocol

To systematically characterize the mechanical response of the SCPC tissue, indenter size, indentation displacement, and loading velocity were introduced as experimental parameters. With regards to indenter size, spherical indenters with radii of 0.25, 0.50, and 1.00 mm were used in this study. For loading velocity, loads of 0.04, 0.06, 0.08, 0.10, 0.15, and 0.20 mm/s were applied to each indenter. The indentation protocol consisted of a single indent with an indentation amplitude of 0.25 mm (i.e., 8.3% of the specimen thickness), which lay within the recommended range of 10% to minimize boundary effects (Garo et al., 2007). The relaxation time was set to 30 s. Data with a displacement error of >5% were discarded. Data were collected at a 100-Hz sampling frequency.

Indentation locations were chosen at random for each specimen. Test order was randomized for each specimen to minimize order effects. The indentation system was operated in displacement control mode during loading. Specimens did not undergo any other preconditioning before experiments. Indentation was done at a velocity of 0.04–0.20 mm/s at six different random sites for each specimen. Between tests, the specimen was allowed to recover for 100 s. Because of the high dissipative nature of neural tissues (Shulyakov et al., 2011), specimens were discarded after indentation. Repetitive tests were performed on 10 different rats.

### Data Analysis

**FIGURE 2:**
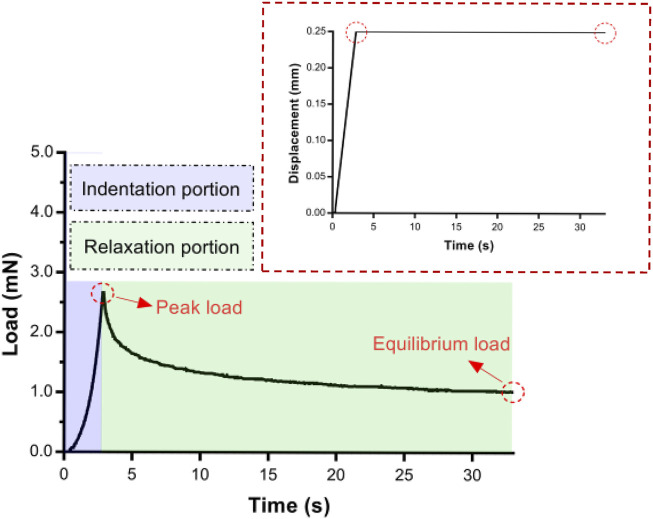
An example of an entire experimental indentation–relaxation load trace for 30 s of 0.50-mm radius indenter. Insert: A typical displacement–time in indentation portion.

Indentation load–relaxation curves consisted of an indentation and a relaxation portion (Figure 2). In the indentation portion, indenters were indented into the tissue at different constant velocities and held during the relaxation portion. Mean peak loads at maximum indentation depth and mean loads at 5, 10, 15, 20, 25, and 30 s during the relaxation phase were calculated to characterize temporal variations.

The elastic modulus (EM) was determined in the form of an indentation method using a spherical indenter. Areas of interest were identified across the surface of each specimen by visual inspection. Indentation load–displacement curves were performed on each region using a Mach-1 Model V500css test device. Indentation parameters were kept constant for each specimen (displacement: 0.25 mm, relaxation time: 30 s). EM at each position was determined by fitting the load–displacement curve with corresponding thickness and an effective Poisson's ratio of 0.5 (Saxena et al., 2012) to an elastic model for indentation as described before (Hayes et al., 1972) (see Eq. 1) using the Mach-1 analysis software (version 6.3, Biomomentum Inc., Laval, QC, Canada). This model is well suited for the mechanical description of specimens bound to flat rigid support (at least 10 times stiffer than the specimen).

$$EM=\frac{P}{H}\times \frac{1-v^{2}}{2ak\left(\frac{a}{h},v\right)}$$ (1)

where *P* = load, *H* = indentation displacement, *ν* = Poisson's ratio, *a* = radius of the contact region, *k* = correction factor dependent on *a*/*h* and *ν*, and *h* = specimen thickness.

### Statistical Analysis

Statistical analyses and graphical representation were done on SPSS version 20.0 (SPSS, Inc., Chicago, IL, USA) or GraphPad Prism version 7.0 (GraphPad Software Inc., San Diego, CA, USA). Differences between the two groups were compared using a two-tailed Student's *t*-test. Non-normally distributed data were compared using the Mann–Whitney–Wilcoxon test. Multiple group comparisons used one-way analysis of variance with Tukey's *post-hoc* test where there was homogeneity of variances or Tamhane *post-hoc* test where variances were unequal. Exact *p*-values are reported in Supplementary Tables S1. *p* ≤ 0.05 indicated statistically significant differences.

## Results

### General Mechanical Response

From the contact point onward, the load recording was stable (Figure 2). The indentation depth was held at a maximum of 0.25 mm (up to peak load), and the load decreased upon relaxation.

### Effect of Velocity

**FIGURE 3:**
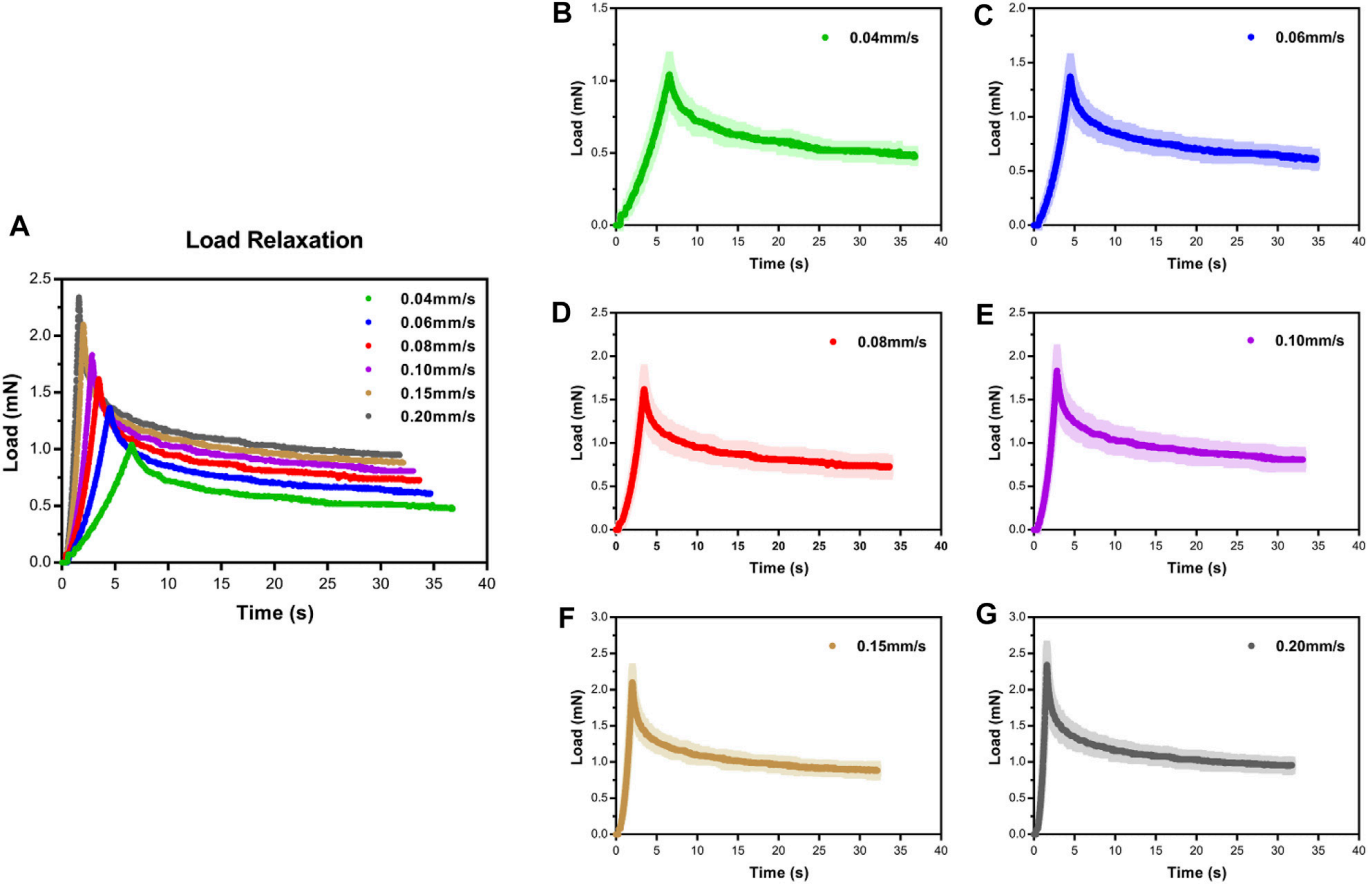
Load–time curves showed loading and relaxation for SCPC tissue up to 0.25-mm displacement at varying velocities using 0.50-mm radius indenter **(A)**. Mean ± standard deviation load–time curves for SCPC indentation experiments **(B–G)**.

**FIGURE 4:**
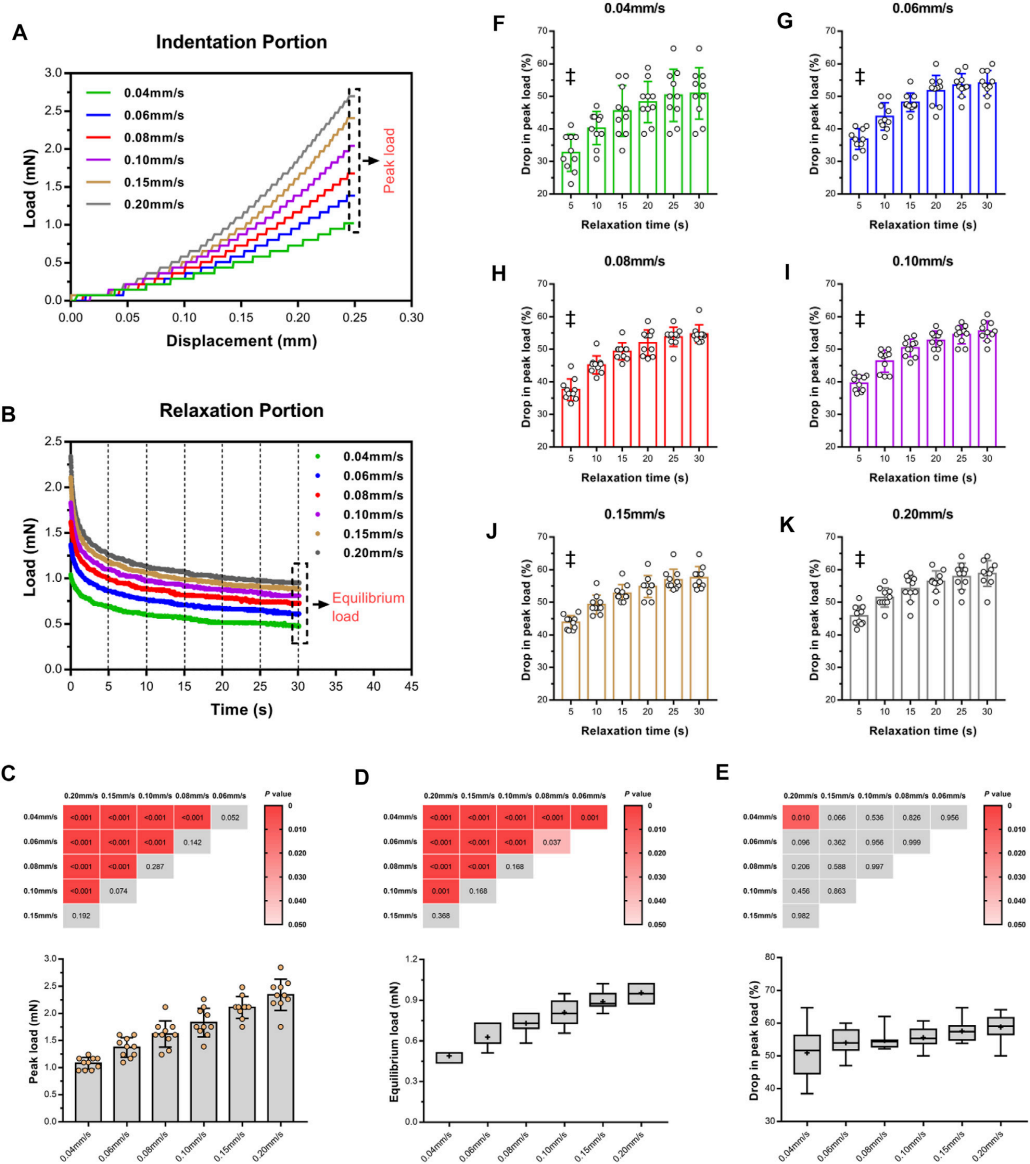
Loading rate sensitivity of *ex vivo* specimens in indentation portion. Indentation loads increased with increasing velocity **(A)**. Isochronal plots of relaxation portion for *ex vivo* specimens at six time points of load–relaxation tests **(B)**. Average peak loads **(C)** and equilibrium loads **(D)** of each test and comparison of difference of two loads **(E)** among varying velocities, inserted with Turkey's *post-hoc* tests. Red block indicate *p* < 0.05, and gray indicated *p* > 0.05. Average relative drop compared to peak load **(F–K)**. ‡ indicate a significant difference (*p* < 0.05) in comparison of 5 s and last two time points (25 and 30 s). Error bars are standard deviation.

To assess the influence of velocity on the cord, we performed a series of single indents at velocities of 0.04–0.20 mm/s. We then analyzed the viscoelastic behavior of the specimen during the indentation load–relaxation period and recorded average load curves at different rates for the 0.50-mm indenter (Figure 3). The mechanical behavior observed during this study was typical of biological materials, with the initial ramp region showing a nonlinear increase in load with applied displacement (taking the form of a “J”), with the load at first being compliant and rising slowly before gradually steepening until maximum applied indentation depth was reached (Figure 4A). Moreover, the ramping slope of higher rates was greater than that of lower rates. As expected for viscoelastic materials, in all conditions, peak load increased with increasing velocity. Relaxation portion data at various loading rates are shown in Figure 4B. The curve showed that most peak load decay occurred in the initial phase (0–5 s). After approximately 10 s, the load converged gradually toward its static equilibrium value. The degree of load relaxation present was approximately 50–60% of the initial peak load over the 30-s period. The peak loads obtained at rates of 0.04–0.20 mm/s were 1.08 ± 0.11, 1.37 ± 0.18, 1.62 ± 0.24, 1.83 ± 0.26, 2.11 ± 0.20, 2.34 ± 0.29 mN, respectively (Figure 4C). Notably, long-term (equilibrium) load after relaxation varied with the loading rate, with higher-rate tests exhibiting larger equilibrium loads at the end of the relaxation period (Figure 4D). Analysis of decay (percentage) between peak and equilibrium load revealed no significant differences at different velocities (Figure 4E).

Figures 4F–K quantitatively display the average drop values in peak load of the specimens at six equidistant time points at various loading rates. At the first isochrone (5 s) examined tests, the specimens showed a significant decay than the last two time points (25 and 30 s) (*p* < 0.001, Supplementary Table S1). In comparison with lower rates, higher rates exhibited a larger drop in peak load for the SCPC tissues at the same isochrones, indicating that the tissues responded more quickly.

Next, we repeated the indentation load–relaxation tests with 0.25- and 1.00-mm indenters at each rate (Supplementary Figures S1,2; Supplementary Tables S2,3). Similar response trends were observed across all indentation tests. To demonstrate the usefulness and applicability of the current data, we further repeated the indentation experiments on the larger animal models (i.e., mature ewes) (N = 6). The results between these two species were similar, suggesting the strong velocity-dependent nature of the SCPC tissue (Supplementary Figure S3).

### Effect of Contact Stress Area

**FIGURE 5:**
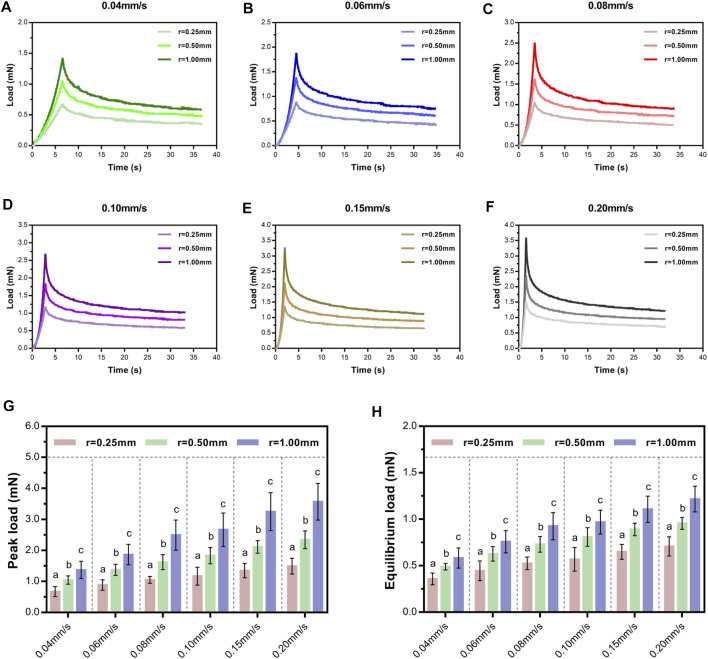
Load–time curves showed loading and relaxation using three indenters of different sizes at varying velocities and held for 30 s **(A–F)**. Comparison of average peak loads **(G)** and equilibrium loads **(H)** of different indenters following a range of rates. Letters indicate significant differences across velocities within a condition with significant indenter-size effects. Error bars are standard deviation.

Here, the variance of contact stress area from common compression factors such as fracture fragment, herniated intervertebral disc, and osteophyte was made by altering the spherical tip radius. Representative indentation and relaxation behaviors of SCPC tissues using spherical indenters with radii of 0.25, 0.5, and 1.00 mm at rates of 0.04–0.20 mm/s are shown in Figures 5A–F. Comparison of peak and equilibrium loads among the three indenters at each rate revealed that load depended on indenter size and significantly increased in magnitude with increasing indenter radius at a constant velocity (*p* ≤ 0.01, Tukey–Kramer multi-comparison test, Figures 5G, H).

**FIGURE 6:**
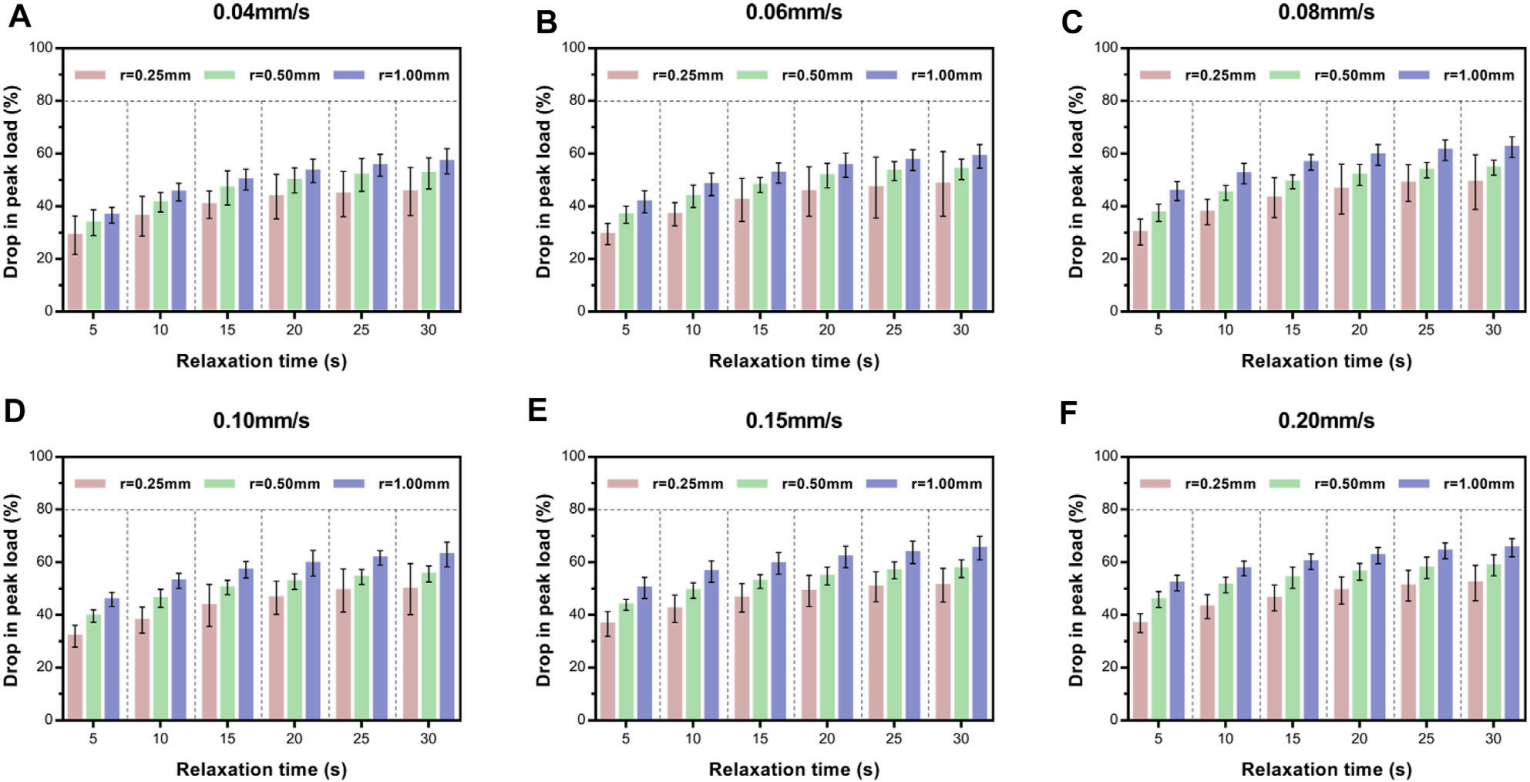
Average relative drop compared with peak load for *ex vivo* specimens at six time points of load–relaxation tests at 0.04 mm/s **(A)**, 0.06 mm/s **(B)**, 0.08 mm/s **(C)**, 0.10 m/s **(D)**, 0.15 m/s **(E)**, and 0.20 mm/s **(F)**. Error bars are standard deviation. For multi-comparison tests, see Supplementary Table S4.

Next, we investigated the effect of indenter size on peak load decay in the relaxation portion (Figure 6). For a given velocity (e.g., 0.04 mm/s), larger contact areas exhibited larger drops in peak load at the same isochrones. Turkey's *post-hoc* tests or Tamhane *post-hoc* tests within each indenter to test the statistical significance of the drop in peak load at different relaxation time points (Supplementary Table S4) revealed that the contact stress area indeed significantly influenced the drop in peak load.

### Elastic Modulus of the Rat Spinal Cord And Pia-Arachnoid Complex

**FIGURE 7:**
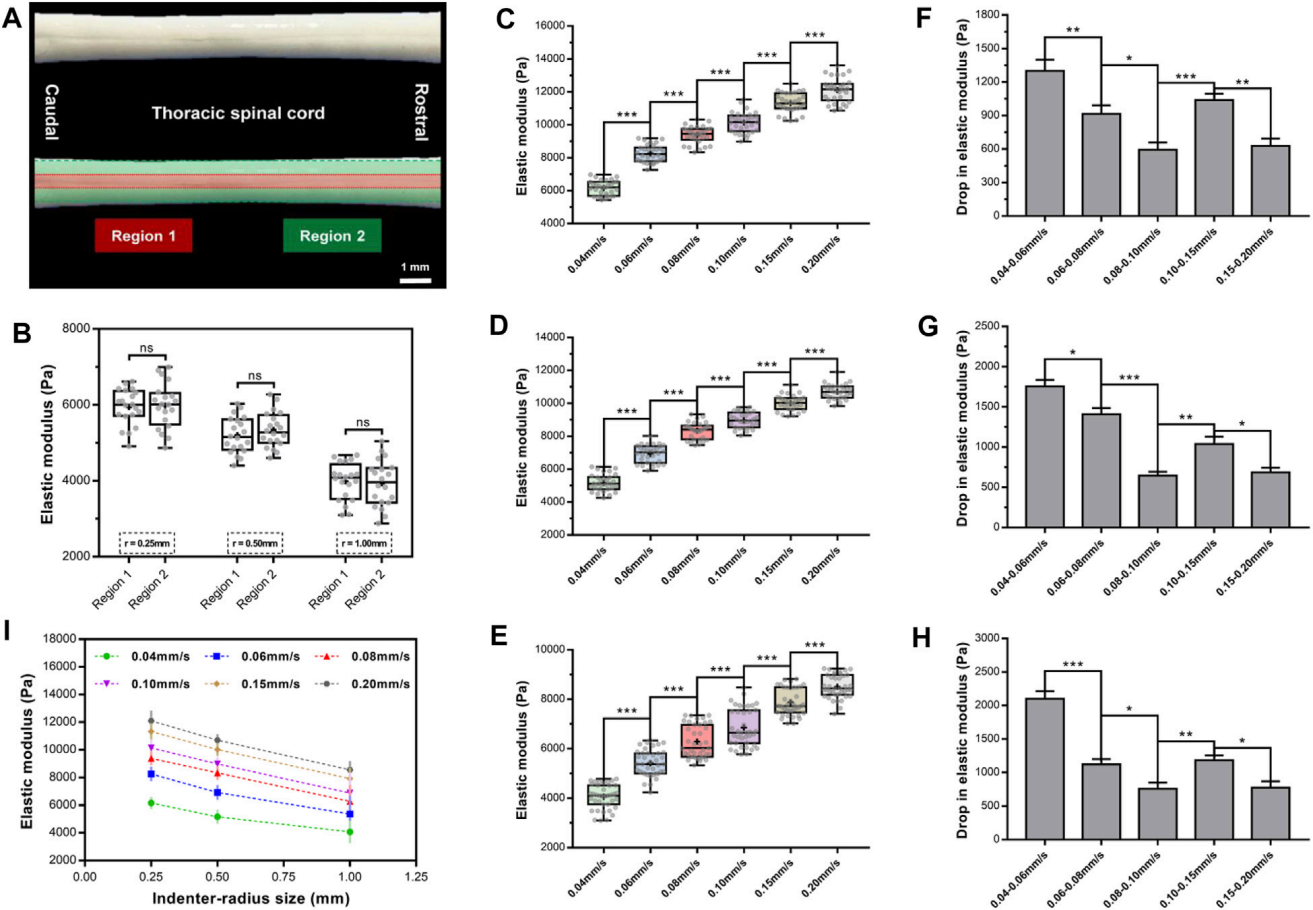
Elastic modulus of rat SCPC tissue under a range of rates and indenters of different sizes. SCPC tissue depicted outlines of region 1 (red) and region 2 (green) **(A)**. There was no significant difference in elastic moduli of two regions (*p* > 0.05, Turkey's *post-hoc* tests) **(B)**. Elastic modulus increased in magnitude with an increase in velocities of indentation for 0.25- **(C)**, 0.50- **(D)**, and 1.00-mm **(E)** radius indenter. A significantly lower drop in elastic modulus than former group either for 0.02 mm/s interval or 0.05 mm/s interval with 0.25- **(F)**, 0.50- **(G),** and 1.00-mm **(H)** radius indenter. Elastic modulus decreased with increasing indenter size at any velocity **(I)**. Error bars are standard error of mean. ^*^ *p* < 0.05; ^**^ *p* < 0.01; ^***^ *p* < 0.001.

We first evaluated the mechanical characteristics and spatial heterogeneity of the elastic properties of rat SCPC tissues (Figure 7A). We defined two tissue regions (Devivo, 2012; Noonan et al., 2012), corresponding to the intermediate zone and marginal zone based on the visual appearance of the ventral surface of the specimen. Inevitably, some diversities were observed for the repetitive experiments due to structural heterogeneity. These differences were not statistically significant (Figure 7B). This indicates that normal SCPC tissue stiffness was spatially relatively homogeneous with low variation in elastic values, which is consistent with past findings (Cooper et al., 2020). The stiffness of regions one and two did not differ significantly across specimens (*p* ≥ 0.05, Supplementary Figure S4, Supplementary Table S5).

To investigate the effect of velocity on EM, we assessed changes in tissue elasticity at 0.04, 0.06, 0.08, 0.10, 0.15, and 0.20 mm/s at an indentation depth of 0.25 mm and averaged mean EM values from 10 force curves, per area. This analysis found that for all three indenters, the EM of the SCPC tissue was significantly depended on indentation velocity and increased in magnitude with increasing indentation velocity (Figures 7C–E, Supplementary Table S6). For the 0.25-mm indenter, at indentation velocities of 0.04–0.20 mm/s, the average EM of SCPC tissue increased from 6,163 to 12,103 Pa, for the 0.50-mm indenter, average EM increased from 5,171 to 10,703 Pa, and for the 1-mm indenter, average EM increased from 4,074 to 8,520 Pa. No inter-animal variability was observed for the EM calculated at each rate (*p* ≥ 0.05, Supplementary Figure S5, Supplementary Table S7). For all three indenters, similar elastic behavior was observed with increasing indentation velocity, which is consistent with previous observations (Budday et al., 2015).

Statistical comparisons were performed of EM within the same interval (0.02 or 0.05 mm/s). The 0.08–0.10 mm/s group exhibited a significantly lower drop in elastic stiffness than the 0.06–0.08 mm/s group, and the 0.08–0.10 mm/s group exhibited a significantly lower drop in elastic stiffness than the 0.04–0.06 mm/s group (*p* ≤ 0.05, Figures 7F–H, Supplementary Table S8). Similarly, the 0.15–0.20 mm/s group exhibited a significantly lower drop in elastic stiffness than the 0.10–0.15 mm/s group (*p* ≤ 0.05, Figures 7F–H, Supplementary Table S8).

Figure 7I shows the effect of indenter size on EM. This analysis found that EM decreased with increasing indenter size. For example, at the rate of 0.04 mm/s and indenter radii of 0.25–1.00 mm, the average EM decreased from 6,163 to 4,074 Pa.

## Discussion

Here, we characterized the mechanical properties of post-mortem rat SCPC tissue *ex vivo*, using indentation testing and quantitatively described mechanical response of the compressed tissue under various loading rates and indenter sizes.

Comparisons between different velocities revealed a strong velocity-dependent nature of SCPC tissue. The indentation tests showed a greater peak load (i.e., stress) upon the spinal cord with the higher initial speed in the ramp or relaxation phase. These results indicate a velocity threshold: speed of impact above that threshold will cause additional damage, whereas speed of impact below that threshold will not be damaging. Increasing speed leading to further injure the cord was consistent with our understanding of tissue mechanics, as more energy could be transferred to the spinal cord, causing damage (Lam et al., 2014). Past studies indicated that damage to neurons and astrocytes occurred at strain rates between 10 and 30/s (Cullen et al., 2007), whereas damage to microtubules occurred at 44/s (Ahmadzadeh et al., 2014).

Interestingly, significant relaxation was observed at all loading rates, with the largest degree of relaxation (i.e., drop in peak load) occurring at the initial 5 s in the relaxation portion. In previous studies, load relaxation behavior has been shown to depend on initial speed, where stress on the cord tissue relaxed more rapidly after a high-rate injury when compared with a slow rate (Carlson et al., 1997a; Carlson et al., 1997b). It is reported that due to the high-velocity nature of the injury, force decreased dramatically to <50% of the peak force within 5–10 s of initial impact (Sjovold et al., 2013), which is consistent with our findings. We inferred that the contribution of this positive mechanical response of the cord might distribute the total load, avoiding local stress concentration. From a clinical standpoint, this suggested that the dynamic behavior of SCPC tissue under high-energy pathological conditions is more sensitive than in low-energy pathological conditions.

Few researchers have investigated the impact of contact stress area on the SCPC tissue. A recent meta-analysis examined several clinically biomechanical factors related to SCI in preclinical studies, attempting to accurately determine effect sizes for each (Batchelor et al., 2013). In that meta-analysis, one of the most important factors identified is compressive pressure, with a power-law distribution being observed between compressive pressure and time to severe neurological injury. In our study, the larger indenter resulted in greater load at the same indentation depth relative to the smaller indenter, indicating that the spinal cord might suffer from a greater degree of external mechanical force and high risk of damage in a similar clinical setting. However, the EM of the SCPC tissue decreased monotonically with increasing tip size, which is consistent with previous findings (Simha et al., 2007). A possible reason for this was its inhomogeneous structure. Assessments at time (∼s) and length scales (∼mm) relevant to cell physiology found that central nervous system tissue was mechanically heterogeneous (Elkin et al., 2007; Christ et al., 2010; Elkin et al., 2011; Iwashita et al., 2014). A recent paper also indicated that stiffness distributions under compression strongly correlated with axon orientation, areas of cell nuclei, and cellular in plane proximity (Koser et al., 2015). Unfortunately, there is no way of avoiding such potential causes from the indentation test data alone. However, the use of multiple indenters may give valuable information on the organization and integrity of the cord, providing a way to relate SCPC properties to tissue microstructure.

Because SCPC tissue is viscoelastic, its modulus (or stiffness) varies under various experimental parameters. However, the influence factors of calculating EM were taken little attention (Bilston and Thibault, 1995; Bilston et al., 2001; Fiford and Bilston, 2005). Whether or not to precondition is controversial, with some researchers suggesting that preconditioning reduces inter-specimen variability (Fallenstein et al., 1969; Bilston et al., 1997; Cheng et al., 2009), whereas others suggest it may confound and influence end modulus measurements due to changes in cord microstructure and different patterns of fluid redistribution in the spinal cord (Ichihara et al., 2001). Thus, researchers must be aware that preconditioning may change tissue architecture and confound conclusions. Another important consideration is the region and orientation of the tested tissue specimen. Here, we did not identify significant differences in stiffness between different regions of the SCPC tissues. Although numerous studies found that gray matter was stiffer than white matter (Prange and Margulies, 2002; Christ et al., 2010; van Dommelen et al., 2010; Koser et al., 2015), others found the opposite (Ozawa et al., 2001; Kruse et al., 2008; Budday et al., 2015), whereas others observed no significant differences in the moduli of white and gray matter (Maikos et al., 2008b; Finan et al., 2012). This was a challenging question to answer because of the high complexity of neural tissue with anisotropic and inhomogeneous mechanical properties (Koser et al., 2015; Cooper et al., 2020). Furthermore, different experimental techniques had unique strengths and limitations and operated at divergent length scales (Budday et al., 2015).

The dura mater is the outermost and most substantial meningeal layer of spinal cord tissue that protects the spinal cord. The rat spinal dura has a modulus in tension that is two orders of magnitude greater than the stiffness of rat spinal cord (Fiford and Bilston, 2005). In animal models of traumatic SCI, the external forces and/or mechanical damages are often transmitted to the dura mater to injure the underlying SCPC tissue. So, the dura will contribute significantly to the overall mechanical response of the spinal cord to traumatic loading and may absorb a large percentage of the kinetic energy (Maikos et al., 2008b). As such, some investigators have suggested removing dura mater to accurately determine how the underlying tissue (i.e., SCPC) responds in the compression stage and the mechanical properties of the SCPC tissue (Clarke et al., 2009; Ramo et al., 2018a). Although the spinal cord tissue is normally not injured from protrusions under the dura in a clinical scenario, the severity of neurological impairment primarily correlates with the degree of substantial damage to the spinal cord parenchyma, according to several previous histopathological studies (Chen et al., 2016). Thus, we believe that it is meaningful and important to directly investigate the dynamic behavior of the SCPC. Additionally, the cord is immersed by cerebrospinal fluid (CSF) that acts as a protecting shock absorber, and it is believed that the response of the CSF cannot be ignored when considering the mechanics of the spinal cord (Persson et al., 2011; Arhiptsov and Marom, 2021). Moreover, without removing the dura, it is experimentally difficult to accurately simulate and measure the *in vivo* CSF pressure in *ex vivo* conditions, limiting the capacity to quantitatively identify the mechanical characteristics of the whole spinal cord tissue. Therefore, we mainly focused on the dynamic behavior of the SCPC without considering the complex effects of dura mater and CSF.

This study has some limitations. First, *ex vivo* analyses do not adequately represent *in vivo* conditions. Even when using optimized storage conditions, CSF pressure and tissue degradation may lead to differences in viscoelastic features in *ex vivo* tissue *versus in vivo* cord tissue (Metz et al., 1970; Etz et al., 2009). However, because the protocols used by these studies are not the same as ours, direct comparisons cannot be made. Future studies will investigate this *in vivo*. Another important limitation is the geometry of the indenter. To ensure accuracy in the comparison of the results calculated from various indenters, we did not use conical or plane-ended cylindrical indenters to simulate the contact stress area of different geometries. However, although the elastic model used to fit the experimental data depended on the geometry of the indenter, the theory of contact mechanics has been well established (McKee et al., 2011). Finally, the 30 s of relaxation might not be enough to identify the long-term relaxation behavior. Future studies will be enhanced by increasing relaxation time to investigate more information on the viscoelastic behavior of the SCPC tissue.

## Conclusion

In conclusion, we comprehensively characterized the regional dynamic behavior of rat SCPC tissue and analyzed the biomechanical effect of velocity and contact stress area on the spinal cord using indentation testing. Our data show that the cord exhibits distinct nonlinear viscoelasticity at <10% of specimen thickness depth magnitudes. At higher velocity and larger contact stress area, the cord withstood a higher peak load and exhibited a more sensitive mechanical relaxation response (i.e., increasing amplitude and speed of drop in peak load), especially the initial phase of residual compression. The cord also became stiffer (i.e., increasing EM) with higher velocity and softer (i.e., decreasing EM) with a larger contact stress area. These findings will improve our understanding of the real-time complex biomechanics involved in traumatic SCI.

## Data Availability

The original contributions presented in the study are included in the article/Supplementary Material, further inquiries can be directed to the corresponding authors.
