# Supplementary material for: Effect of Velocity and Contact Stress Area on the Dynamic Behavior of the Spinal Cord Under Different Testing Conditions
Source: Front Bioeng Biotechnol. 2022 Mar 4;10:762555. doi: 10.3389/fbioe.2022.762555 (PMC8931460; doi:10.3389/fbioe.2022.762555)
Supplement: Supplementary file 2 [file DataSheet4.PDF]

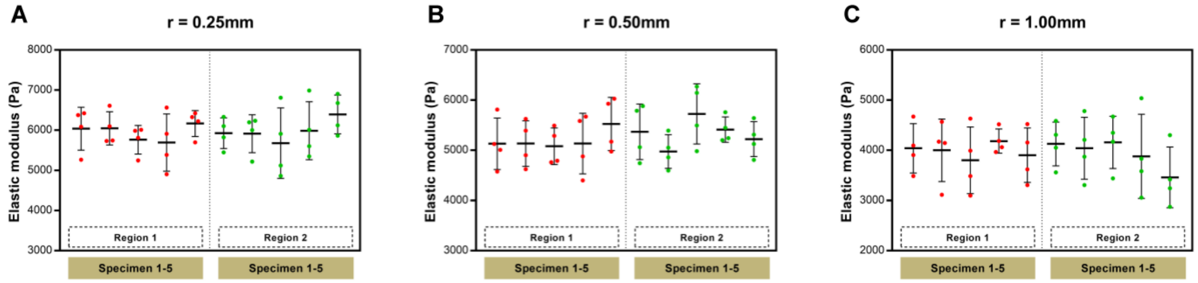

Supplementary Figure 4. Elastic modulus of the SCPC tissue presented by each animal (specimen 1-5) and differentiating region 1 and region 2. Each point is the averaged elastic modulus value obtained per analyzed area with 0.50 mm indenter at the rate of 0.04 mm/s. For multi-comparison tests see Supplementary Table 5.
