# Supplementary material for: Effect of Velocity and Contact Stress Area on the Dynamic Behavior of the Spinal Cord Under Different Testing Conditions
Source: Front Bioeng Biotechnol. 2022 Mar 4;10:762555. doi: 10.3389/fbioe.2022.762555 (PMC8931460; doi:10.3389/fbioe.2022.762555)
Supplement: Supplementary file 3 [file Table1.pdf]

Supplementary Table 1. Statistical comparison of drop in peak load among six equidistant time points in the relaxation portion (indenter radius = 0.50 mm).

Velocity	0.04mm/s					0.06mm/s					0.08mm/s				
**Relaxation time**	**10s**	**15s**	**20s**	**25s**	**30s**	**10s**	**15s**	**20s**	**25s**	**30s**	**10s**	**15s**	**20s**	**25s**	**30s**
**5s**	0.159	0.002	0.002	<0.001	<0.001	0.002	<0.001	<0.001	<0.001	<0.001	<0.001	<0.001	<0.001	<0.001	<0.001
**10s**		0.526	0.526	0.113	0.021		0.128	<0.001	<0.001	<0.001		0.046	<0.001	<0.001	<0.001
**15s**			1.000	0.947	0.626			0.298	0.034	0.012			0.411	0.025	0.004
**20s**				0.947	0.626				0.916	0.736				0.777	0.365
**25s**					0.984					0.999					0.984
**Velocity**	**0.10mm/s**					**0.15mm/s**					**0.20mm/s**				
**Relaxation time**	**10s**	**15s**	**20s**	**25s**	**30s**	**10s**	**15s**	**20s**	**25s**	**30s**	**10s**	**15s**	**20s**	**25s**	**30s**
**5s**	<0.001	<0.001	<0.001	<0.001	<0.001	0.002	<0.001	<0.001	<0.001	<0.001	0.012	<0.001	<0.001	<0.001	<0.001
**10s**		0.033	<0.001	<0.001	<0.001		0.109	0.002	<0.001	<0.001		0.531	0.035	0.002	<0.001
**15s**			0.496	0.031	0.003			0.649	0.034	0.008			0.737	0.203	0.056
**20s**				0.741	0.246				0.617	0.301				0.935	0.652
**25s**					0.958					0.995					0.992

Supplementary Table 2. Statistical comparison of drop in peak load among six equidistant time points in the relaxation portion (indenter radius = 0.25 mm).

Velocity	0.04mm/s					0.06mm/s					0.08mm/s				
Relaxation time	10s	15s	20s	25s	30s	10s	15s	20s	25s	30s	10s	15s	20s	25s	30s
5s	0.323	0.018	0.001	<0.001	<0.001	0.413	0.022	0.002	<0.001	<0.001	0.239	0.005	<0.001	<0.001	<0.001
10s		0.799	0.284	0.167	0.095		0.749	0.269	0.130	0.058		0.615	0.130	0.026	0.020
15s			0.953	0.867	0.720			0.965	0.845	0.647			0.927	0.581	0.526
20s				1.000	0.994				0.999	0.978				0.985	0.974
25s					1.000					0.999					1.000
Velocity	0.10mm/s					0.15mm/s					0.20mm/s				
Relaxation time	10s	15s	20s	25s	30s	10s	15s	20s	25s	30s	10s	15s	20s	25s	30s
5s	0.404	0.007	<0.001	<0.001	<0.001	0.208	0.003	<0.001	<0.001	<0.001	0.096	0.002	<0.001	<0.001	<0.001
10s		0.508	0.096	0.011	0.007		0.546	0.086	0.017	0.009		0.725	0.109	0.015	0.004
15s			0.936	0.491	0.396			0.901	0.552	0.413			0.825	0.350	0.158
20s				0.958	0.917				0.988	0.955				0.968	0.820
25s					1.000					1.000					0.998

Supplementary Table 3. Statistical comparison of drop in peak load among six equidistant time points in the relaxation portion (indenter radius = 1.00 mm).

**Velocity**	**0.04mm/s**					**0.06mm/s**					**0.08mm/s**				
**Relaxation time**	**10s**	**15s**	**20s**	**25s**	**30s**	**10s**	**15s**	**20s**	**25s**	**30s**	**10s**	**15s**	**20s**	**25s**	**30s**
**5**	<0.001	<0.001	<0.001	<0.001	<0.001	0.012	<0.001	<0.001	<0.001	<0.001	0.002	<0.001	<0.001	<0.001	<0.001
**10**		0.092	<0.001	<0.001	<0.001		0.206	0.004	<0.001	<0.001		0.130	0.001	<0.001	<0.001
**15**			0.451	0.042	0.004			0.654	0.127	0.019			0.532	0.072	0.011
**20**				0.840	0.334				0.904	0.467				0.880	0.473
**25**					0.956					0.970					0.981
**Velocity**	**0.10mm/s**					**0.15mm/s**					**0.20mm/s**				
**Relaxation time**	**10s**	**15s**	**20s**	**25s**	**30s**	**10s**	**15s**	**20s**	**25s**	**30s**	**10s**	**15s**	**20s**	**25s**	**30s**
**5s**	0.001	<0.001	<0.001	<0.001	<0.001	0.017	<0.001	<0.001	<0.001	<0.001	0.002	<0.001	<0.001	<0.001	<0.001
**10s**		0.110	0.001	<0.001	<0.001		0.551	0.043	0.003	<0.001		0.403	0.009	<0.001	<0.001
**15s**			0.624	0.070	0.008			0.764	0.230	0.032			0.564	0.044	0.004
**20s**				0.809	0.319				0.939	0.479				0.756	0.248
**25s**					0.963					0.952					0.953

Supplementary Table 4. Statistical comparison of drop in peak load among three indenters at six equidistant time points.

Velocity	0.04mm/s											
Relaxation time	5s		10s		15s		20s		25s		30s	
Indenter	0.50mm	1.00mm	0.50mm	1.00mm	0.50mm	1.00mm	0.50mm	1.00mm	0.50mm	1.00mm	0.50mm	1.00mm
0.25mm	0.141	0.010	0.077	0.002	0.033	0.001	0.084	0.004	0.052	0.003	0.080	0.002
0.50mm		0.462		0.245		0.377		0.408		0.445		0.313

Velocity	0.06mm/s											
Relaxation time	5s		10s		15s		20s		25s		30s	
Indenter	0.50mm	1.00mm	0.50mm	1.00mm	0.50mm	1.00mm	0.50mm	1.00mm	0.50mm	1.00mm	0.50mm	1.00mm
0.25mm	0.001	<0.001	0.004	<0.001	0.068	0.001	0.122	0.007	0.157	0.010	0.274	0.016
0.50mm		0.022		0.064		0.163		0.402		0.430		0.348

Velocity	0.08mm/s											
Relaxation time	5s		10s		15s		20s		25s		30s	
Indenter	0.50mm	1.00mm	0.50mm	1.00mm	0.50mm	1.00mm	0.50mm	1.00mm	0.50mm	1.00mm	0.50mm	1.00mm
0.25mm	0.001	<0.001	0.001	<0.001	0.029	<0.001	0.161	<0.001	0.085	<0.001	0.163	<0.001
0.50mm		<0.001		0.001		0.007		0.033		0.005		0.034

Velocity	0.10mm/s											
Relaxation time	5s		10s		15s		20s		25s		30s	
Indenter	0.50mm	1.00mm	0.50mm	1.00mm	0.50mm	1.00mm	0.50mm	1.00mm	0.50mm	1.00mm	0.50mm	1.00mm
0.25mm	<0.001	<0.001	<0.001	<0.001	0.019	<0.001	0.026	<0.001	0.088	<0.001	0.133	<0.001
0.50mm		<0.001		0.002		0.019		0.010		0.013		0.041

Velocity	0.15mm/s											
Relaxation time	5s		10s		15s		20s		25s		30s	
Indenter	0.50mm	1.00mm	0.50mm	1.00mm	0.50mm	1.00mm	0.50mm	1.00mm	0.50mm	1.00mm	0.50mm	1.00mm
0.25mm	0.001	<0.001	0.002	<0.001	0.008	<0.001	0.027	<0.001	0.013	<0.001	0.021	<0.001
0.50mm		0.002		0.002		0.003		0.004		0.005		0.004

Velocity	0.20mm/s											
Relaxation time	5s		10s		15s		20s		25s		30s	
Indenter	0.50mm	1.00mm	0.50mm	1.00mm	0.50mm	1.00mm	0.50mm	1.00mm	0.50mm	1.00mm	0.50mm	1.00mm
0.25mm	<0.001	<0.001	<0.001	<0.001	0.001	<0.001	0.001	<0.001	0.006	<0.001	0.051	<0.001
0.50mm		<0.001		0.001		0.007		0.005		0.009		0.002

Supplementary Table 5. Statistical comparison of the rat SCPC tissue stiffness between each animal.

Indenter	0.25mm							
	Region 1				Region 2			
Specimen	#2	#3	#4	#5	#2	#3	#4	#5
#1	1.000	0.929	0.854	0.995	1.000	0.978	1.000	0.817
#2		0.921	0.842	0.996		0.981	1.000	0.804
#3			1.000	0.769			0.951	0.495
#4				0.655				0.880

Indenter	0.50mm							
	Region 1				Region 2			
Specimen	#2	#3	#4	#5	#2	#3	#4	#5
#1	1.000	1.000	1.000	0.796	0.715	0.782	1.000	0.989
#2		1.000	1.000	0.802		0.166	0.635	0.929
#3			1.000	0.722			0.849	0.511
#4				0.800				0.971

Indenter	1.00mm							
	Region 1				Region 2			
Specimen	#2	#3	#4	#5	#2	#3	#4	#5
#1	1.000	0.968	0.995	0.996	1.000	1.000	0.978	0.563
#2		0.983	0.988	0.999		0.999	0.995	0.676
#3			0.846	0.999			0.966	0.520
#4				0.942				0.869

Supplementary Table 6. Statistical comparison of the elastic moduli of the SCPC tissue among varying velocities.

**Indenter**			**0.25mm**		
**Velocity**	**0.06mm/s**	**0.08mm/s**	**0.10mm/s**	**0.15mm/s**	**0.20mm/s**
**0.04mm/s**	<0.001	<0.001	<0.001	<0.001	<0.001
**0.06mm/s**		<0.001	<0.001	<0.001	<0.001
**0.08mm/s**			<0.001	<0.001	<0.001
**0.10mm/s**				<0.001	<0.001
**0.15mm/s**					<0.001
**Indenter**			**0.50mm**		
**Velocity**	**0.06mm/s**	**0.08mm/s**	**0.10mm/s**	**0.15mm/s**	**0.20mm/s**
**0.04mm/s**	<0.001	<0.001	<0.001	<0.001	<0.001
**0.06mm/s**		<0.001	<0.001	<0.001	<0.001
**0.08mm/s**			<0.001	<0.001	<0.001
**0.10mm/s**				<0.001	<0.001
**0.15mm/s**					<0.001
**Indenter**			**1.00mm**		
**Velocity**	**0.06mm/s**	**0.08mm/s**	**0.10mm/s**	**0.15mm/s**	**0.20mm/s**
**0.04mm/s**	<0.001	<0.001	<0.001	<0.001	<0.001
**0.06mm/s**		<0.001	<0.001	<0.001	<0.001
**0.08mm/s**			<0.001	0.015	<0.001
**0.10mm/s**				<0.001	<0.001
**0.15mm/s**					<0.001

Supplementary Table 7. Statistical comparison of elastic moduli of the SCPC tissue of each animal (specimen 1-5).

**Velocity**						**0.04mm/s**						
**Indenter**		**0.25mm**				**0.50mm**				**1.00mm**		
**Specimen**	**#2**	**#3**	**#4**	**#5**	**#2**	**#3**	**#4**	**#5**	**#2**	**#3**	**#4**	**#5**
**#1**	0.922	0.995	1.000	0.991	0.924	0.998	0.999	1.000	0.519	0.911	1.000	0.998
**#2**		0.746	0.892	0.715		0.985	0.825	0.847		0.947	0.650	0.708
**#3**			0.998	1.000			0.981	0.987			0.967	0.982
**#4**				0.996				1.000				1.000

**Velocity**						**0.06mm/s**						
**Indenter**		**0.25mm**				**0.50mm**				**1.00mm**		
**Specimen**	**#2**	**#3**	**#4**	**#5**	**#2**	**#3**	**#4**	**#5**	**#2**	**#3**	**#4**	**#5**
**#1**	0.990	0.701	0.188	1.000	0.918	1.000	0.724	1.000	0.971	0.999	0.998	0.996
**#2**		0.919	0.389	0.985		0.928	0.260	0.836		0.906	0.998	0.862
**#3**			0.860	0.669			0.706	0.999			0.980	1.000
**#4**				0.170				0.833				0.962

**Velocity**						**0.08mm/s**						
**Indenter**		**0.25mm**				**0.50mm**				**1.00mm**		
**Specimen**	**#2**	**#3**	**#4**	**#5**	**#2**	**#3**	**#4**	**#5**	**#2**	**#3**	**#4**	**#5**
**#1**	0.975	0.971	0.854	0.959	0.945	0.667	0.922	0.832	0.989	1.000	1.000	1.000
**#2**		0.747	0.517	1.000		0.973	1.000	0.998		0.994	0.985	0.982
**#3**			0.995	0.698			0.984	0.998			1.000	1.000
**#4**				0.466				0.999				1.000

**Velocity**						**0.10mm/s**						
**Indenter**		**0.25mm**				**0.50mm**				**1.00mm**		
**Specimen**	**#2**	**#3**	**#4**	**#5**	**#2**	**#3**	**#4**	**#5**	**#2**	**#3**	**#4**	**#5**
**#1**	0.828	0.976	0.945	0.717	0.951	0.343	0.788	0.924	1.000	0.991	0.999	1.000
**#2**		0.990	0.998	1.000		0.760	0.975	1.000		0.983	0.997	1.000
**#3**			1.000	0.961			0.975	0.812			1.000	0.997
**#4**				0.985				0.987				1.000

**Velocity**						**0.15mm/s**						
**Indenter**		**0.25mm**				**0.50mm**				**1.00mm**		
**Specimen**	**#2**	**#3**	**#4**	**#5**	**#2**	**#3**	**#4**	**#5**	**#2**	**#3**	**#4**	**#5**
**#1**	0.614	0.634	0.965	0.661	0.977	0.936	0.747	0.572	0.995	0.954	0.999	0.981
**#2**		1.000	0.933	1.000		1.000	0.969	0.887		0.808	0.961	0.876
**#3**			0.942	1.000			0.992	0.948			0.993	1.000
**#4**				0.953				0.998				0.999

**Velocity**						**0.20mm/s**						
**Indenter**		**0.25mm**				**0.50mm**				**1.00mm**		
**Specimen**	**#2**	**#3**	**#4**	**#5**	**#2**	**#3**	**#4**	**#5**	**#2**	**#3**	**#4**	**#5**
**#1**	0.824	0.916	1.000	0.684	0.989	0.996	1.000	0.982	0.999	0.986	0.773	1.000
**#2**		0.999	0.838	0.999		0.914	0.982	0.845		0.998	0.876	1.000
**#3**			0.925	0.989			0.998	1.000			0.965	0.996
**#4**				0.701				0.989				0.841

Supplementary Table 8. Statistical comparison of the difference of elastic moduli of the SCPC tissue.

**Indenter**			**0.25mm**	
**Velocity interval**	**0.06-0.08mm/s**	**0.08-0.10mm/s**	**0.10-0.15mm/s**	**0.15-0.20mm/s**
**0.04-0.06mm/s**	<0.001	<0.001	<0.001	<0.001
**0.06-0.08mm/s**		0.041	1.000	0.067
**0.08-0.10mm/s**			0.008	1.000
**0.10-0.15mm/s**				0.014
**Indenter**			**0.50mm**	
**Velocity interval**	**0.06-0.08mm/s**	**0.08-0.10mm/s**	**0.10-0.15mm/s**	**0.15-0.20mm/s**
**0.04-0.06mm/s**	0.034	<0.001	<0.001	<0.001
**0.06-0.08mm/s**		<0.001	0.032	<0.001
**0.08-0.10mm/s**			0.005	1.000
**0.10-0.15mm/s**				0.023
**Indenter**			**1.00mm**	
**Velocity interval**	**0.06-0.08mm/s**	**0.08-0.10mm/s**	**0.10-0.15mm/s**	**0.15-0.20mm/s**
**0.04-0.06mm/s**	0.003	<0.001	0.093	<0.001
**0.06-0.08mm/s**		0.021	0.771	0.052
**0.08-0.10mm/s**			<0.001	0.997
**0.10-0.15mm/s**				0.001
