# Supplementary material for: Effect of Velocity and Contact Stress Area on the Dynamic Behavior of the Spinal Cord Under Different Testing Conditions
Source: Front Bioeng Biotechnol. 2022 Mar 4;10:762555. doi: 10.3389/fbioe.2022.762555 (PMC8931460; doi:10.3389/fbioe.2022.762555)
Supplement: Supplementary file 4 [file DataSheet3.PDF]

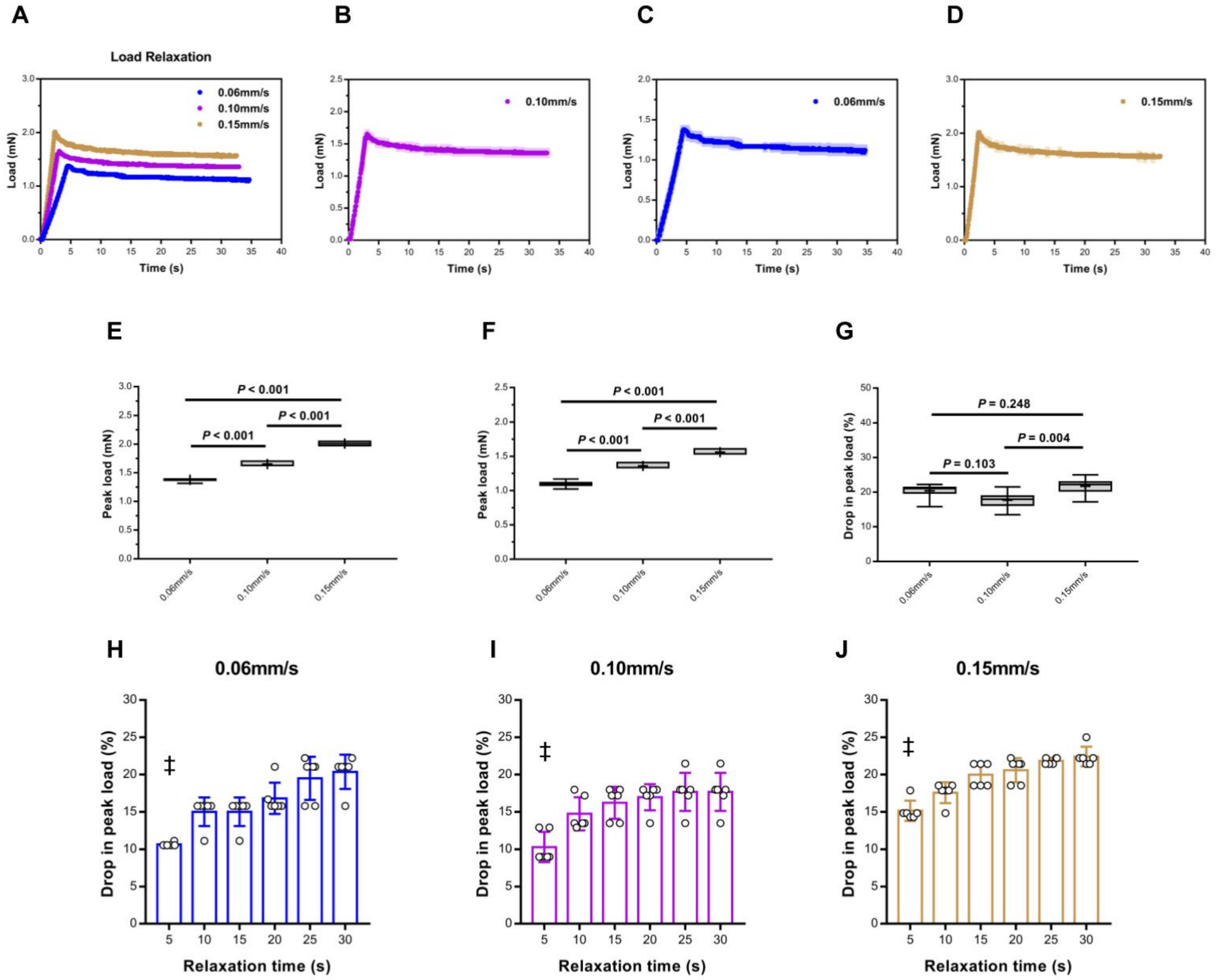


**Supplementary Figure 3. (A)** Load-time curves showing the loading and relaxation for the ewes SCPC tissue up to 0.25 mm displacement at varying velocities using 0.50 mm radius indenter. **(B-D)** Mean ± standard deviation load-time curves for the SCPC indentation experiments. **(E-G)** The average peak loads and equilibrium loads of each test and the comparison of the difference of the two loads among varying velocities. **(H-J)** The average relative drop compared to the peak load. ‡ indicate a significant difference ($P < 0.05$) in the comparison of 5 s and the last two time points (25 s and 30 s).
