# Supplementary material for: Effect of Velocity and Contact Stress Area on the Dynamic Behavior of the Spinal Cord Under Different Testing Conditions
Source: Front Bioeng Biotechnol. 2022 Mar 4;10:762555. doi: 10.3389/fbioe.2022.762555 (PMC8931460; doi:10.3389/fbioe.2022.762555)
Supplement: Supplementary file 5 [file DataSheet1.PDF]

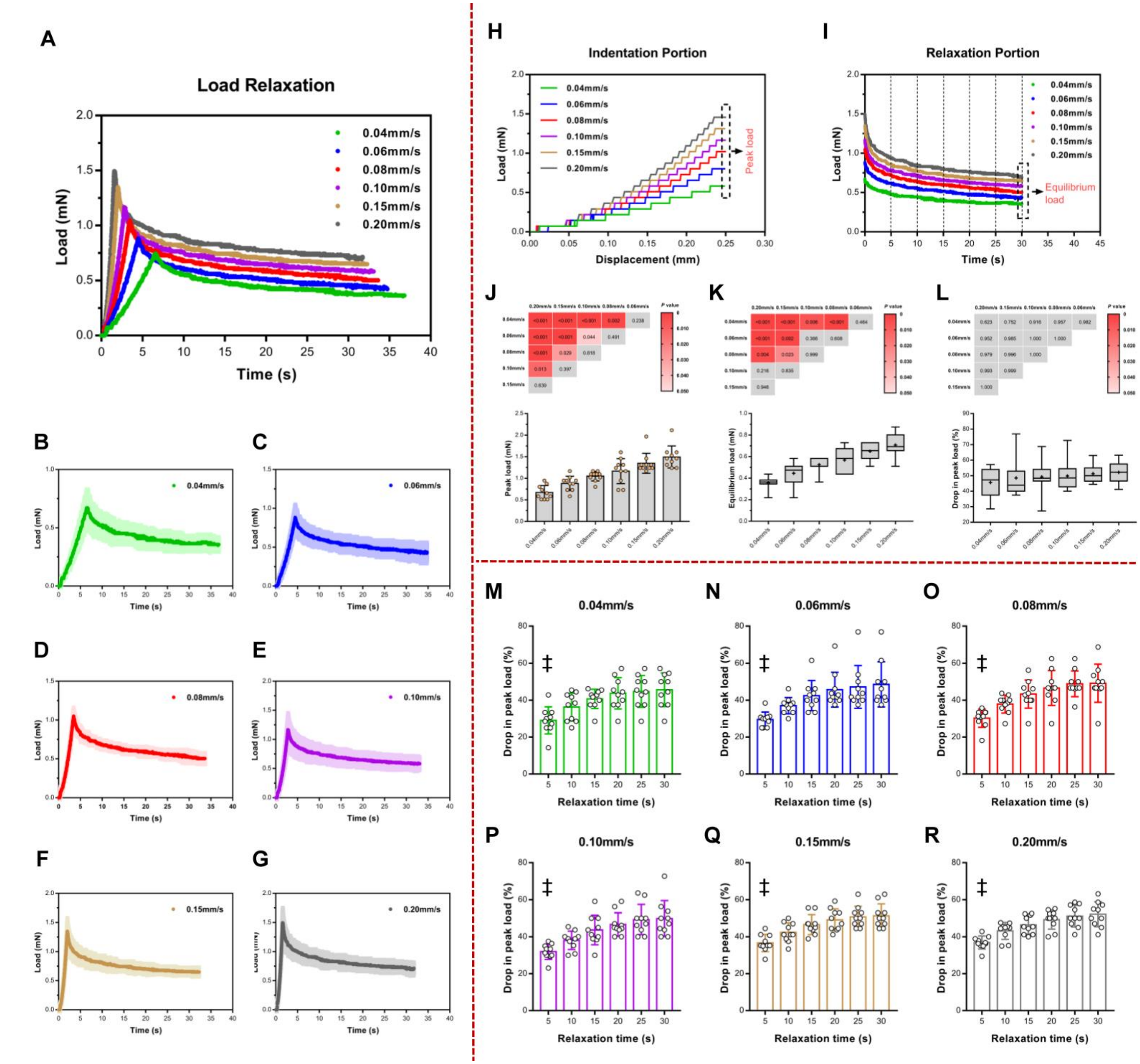

Supplementary Figure 1. Load-time curves showed the loading and relaxation for the SCPC tissue up to 0.25 mm displacement at varying velocities using 0.25 mm radius indenter **(A)**. Mean ± standard deviation load-time curves for the SCPC indentation experiments **(B-G)**. Loading rate sensitivity of *ex vivo* specimens in the indentation portion. Indentation loads increase with increasing velocity **(H)**. Isochronal plots of the relaxation portion for *ex vivo* specimens at 6 time points of the load-relaxation tests **(I)**. The average peak loads **(J)** and equilibrium loads **(K)** of each test and the comparison of the difference of the two loads **(L)** among varying velocities, inserted with Turkey's *post hoc* tests. Red block indicate $P < 0.05$, and gray indicated $P > 0.05$. The average relative drop compared to the peak load **(M-R)**. ‡ indicate a significant difference ($P < 0.05$) in the comparison of 5 s and the last two time points (25 s and 30 s).
