# Supplementary material for: Effect of Velocity and Contact Stress Area on the Dynamic Behavior of the Spinal Cord Under Different Testing Conditions
Source: Front Bioeng Biotechnol. 2022 Mar 4;10:762555. doi: 10.3389/fbioe.2022.762555 (PMC8931460; doi:10.3389/fbioe.2022.762555)
Supplement: Supplementary file 6 [file DataSheet5.PDF]

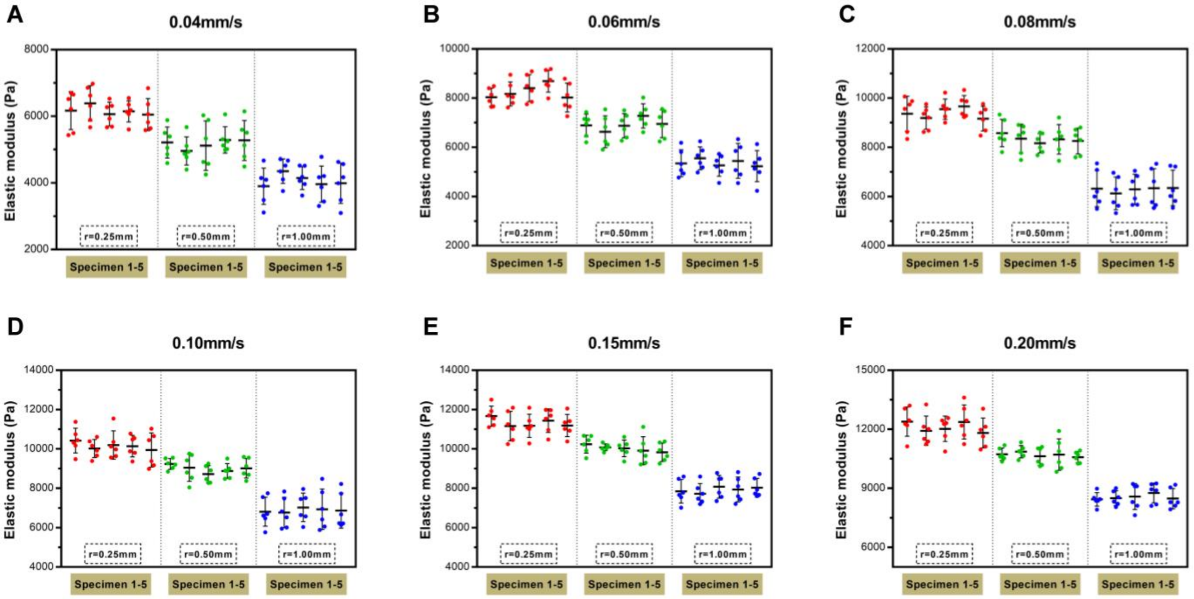

Supplementary Figure 5. Comparison of the elastic moduli of the SCPC tissue of each animal (specimen 1-5) with indenter of different sizes. Inter-animal variability was low, and the elastic moduli were statistically similar in all animals at the same velocity with an indenter of the same size. Moreover, the average elastic moduli decreased with the increasing indenter size in all animals at the same velocity. For multi-comparison tests see Supplementary Table 7.
